# Prospective Assessment of Fluorine-18-Fluorodeoxyglucose-Positron Emission Tomography/Computed Tomography (FDG-PET/CT) for Early Identification of Checkpoint-Inhibitor-Induced Pseudoprogression

**DOI:** 10.3390/cancers16050964

**Published:** 2024-02-27

**Authors:** Sif Homburg, Charlotte Birk Christensen, Magnus Pedersen, Simon Grund Sørensen, Marco Donia, Inge Marie Svane, Helle Westergren Hendel, Eva Ellebaek

**Affiliations:** 1 Department of Oncology, Copenhagen University Hospital, Herlev and Gentofte, 2730 Herlev, Denmark; sifhomburg@hotmail.com; 2 Department of Clinical Physiology and Nuclear Medicine, Copenhagen University Hospital, Herlev and Gentofte, 2730 Herlev, Denmark; 3 National Center for Cancer Immune Therapy (CCIT-DK), Department of Oncology, Copenhagen University Hospital, Herlev and Gentofte, 2730 Herlev, Denmark; 4 Department of Molecular Medicine, Aarhus University Hospital, 8000 Aarhus, Denmark

**Keywords:** metastatic melanoma, immune checkpoint inhibitors, FDG-PET/CT, dual-time point FDG-PET/CT, PERCIMT, pseudoprogression

## Abstract

**Simple Summary:**

Immune checkpoint inhibitors are standard treatment for patients with metastatic melanoma, and treatment response is most optimally evaluated with FDG-PET/CT. Pseudoprogression describes the difficulty of distinguishing between real cancer growth and growth occurring due to active infiltrating immune cells that mistakenly look like tumor growth on scans. The aim of our study was to investigate whether dual-time point (DTP) FDG-PET/CT scan and modified response criteria (PERCIMT) could be helpful in detecting pseudoprogression. Our findings suggest limited efficacy of DTP FDG-PET/CT whereas PERCIMT criteria could be included in the clinical decision making ensuring correct treatment choices.

**Abstract:**

The activity of immune checkpoint inhibitors (ICIs) in patients with metastatic melanoma is often monitored using fluorine-18-fluorodeoxyglucose-positron emission tomography/computed tomography (FDG-PET/CT) scans. However, distinguishing disease progression (PD) from pseudoprogression (PsPD), where increased FDG uptake might reflect immune cell activity rather than tumor growth, remains a challenge. This prospective study compared the efficacy of dual-time point (DTP) FDG-PET/CT with modified response criteria (PERCIMT) in differentiating PsPD from PD. From July 2017–January 2021, 41 patients suspected to have PsPD on an evaluation scan were prospectively included (29 evaluable). A subsequent DTP FDG-PET/CT scan was conducted within 14 days, followed by a confirmatory FDG-PET/CT scan. Additionally, PERCIMT were applied. DTP FDG-PET/CT identified 24% with PsPD and 76% with PD. Applying PERCIMT criteria, 69% showed PsPD, while 31% had PD. On follow-up, 10 patients (34%) demonstrated confirmed PsPD, while 19 (66%) exhibited PD. The sensitivity and specificity of DTP FDG-PET/CT were 20% and 74%, respectively, and for PERCIMT this was 80% and 37%, respectively. Our findings suggest limited efficacy of DTP FDG-PET/CT in distinguishing PsPD from PD in ICI-treated patients with metastatic melanoma. The use of PERCIMT could complement clinical assessment and be incorporated in multidisciplinary team conferences for enhanced decision-making.

## 1. Introduction

Immune checkpoint inhibitors (ICIs) have been standard treatment for patients with metastatic melanoma for more than a decade and have significantly improved overall survival (OS) in the real-world [1,2].

In the first clinical studies with ICIs, a fraction of patients with metastatic melanoma had early radiological evidence of disease progression (PD) on fluorine-18-fluorodeoxyglucose-positron emission tomography (FDG-PET)/computed tomography (CT), but ultimately achieved an objective response. This phenomenon was later named pseudoprogression (pseudoprogressive disease, PsPD) and describes apparent PD (increase in size and/or FDG-avidity of pre-existing lesions or the presence of new tumor lesions) on an evaluation scan, subsequently followed by a decrease in tumor burden. ICI-related PsPD is observed in 10–15% of patients with melanomas. However, most patients with initial progression will have true PD and not PsPD [3,4,5]. Tools for early identification of true PsPD could greatly improve the management of patients treated with ICI, leading to timely discontinuation, reduced toxicity, and a possible shift in treatment. FDG-PET-CT is an accurate, non-invasive imaging modality for patients with melanoma because the cellular glucose metabolism is highly increased in these tumors. A meta-analysis including 105.228 patients estimated the diagnostic sensitivity and specificity of FDG-PET/CT in detecting distant metastasis from melanoma to be 86% and 91%, respectively, and FDG-PET/CT is widely used in the screening for distant metastases [6] as well as to monitor treatment response in patients treated for metastatic melanoma. However, FDG is not a cancer-specific tracer. Activation of the immune system elicited by ICI can lead to increased glycolytic use by the immune cells infiltrating the tumor, and thereby increased FDG uptake is not only observed in the tumor cells but also in the tumor infiltrating immune cells and in the tumor microenvironment. Therefore, there is a need to identify and develop additional tools and molecular imaging strategies that can differentiate metabolically active cancer cells from activated immune cells when evaluating tumor response in patients with melanoma receiving ICI [7].

Dual-time point (DTP) imaging and the development of more dedicated and robust response criteria have been proposed to separate patients with true clinical benefit, including patients with PsPD, from those with PD. The use of DTP FDG-PET/CT is based on results from previous studies demonstrating that the accumulation of FDG continues to increase in malignant tumors for several hours after the injection of the radiotracer, whereas FDG accumulation decreases or remains stable in inflammatory cells. Therefore, the difference in the time course of FDG uptake could potentially be used to improve the accuracy of the scans to differentiate benign and malignant lesions [7,8,9]. DTP FDG-PET/CT has previously been investigated to potentially enhance the diagnostic specificity and accuracy of FDG-PET/CT to differentiate malignant from benign lesions [10,11].

Applying conventional morphologic and metabolic response criteria might lead to the underestimation of response to ICI, and thereby premature treatment withdrawal. To avoid false negative results to treatment, modified response criteria have been proposed to increase the diagnostic accuracy of FDG-PET in treatment monitoring of ICI [12,13]. Prior studies have investigated the use of single time point FDG-PET/CT for early detection of PsPD [13,14].

To our knowledge, no studies have previously reported on early identification of PsPD in metastatic melanoma using DTP FDG-PET/CT. In this study, we have assessed whether DTP FDG-PET/CT increases the sensitivity and specificity of FDG-PET/CT and is able to differentiate metabolic-active cancer cells from activated immune cells in patients with metastatic melanoma treated with ICI, and thereby distinguish PsPD from PD. Moreover, we explore the potential of applying the PET Response Evaluation Criteria for Immunotherapy (PERCIMT) [12] to aid in discerning PsPD from actual PD. PERCIMT is a modified PET-based metabolic criteria, that appear to provide an accurate assessment of metabolic response that correlates with clinical response and overall patient survival [15,16]. This investigation could help optimize present clinical strategies and improve the accuracy of treatment response assessment in the context of metastatic melanoma during ICI therapy.

## 2. Materials and Methods

### 2.1. Patients

This is a prospective single-center study including patients with metastatic melanoma who were treated with ICI and suspected to have PsPD on routine FDG-PET/CT scans. Suspected PsPD was defined as visually assessed increased FDG-metabolism in pre-existing lesions, increased number of lesions, and/or increase in size of FDG-avid lesions without any clinical signs of progression. The only exclusion criterion was other active malignancies.

Baseline data were extracted from the patients’ files and included disease stage, localization of metastatic sites, BRAF mutation status, performance status, lactate dehydrogenase, and current treatment with ICI, as well as potential previous treatments with ICI.

The study was conducted in accordance with the Declaration of Helsinki and with Good Clinical Practice as defined by the International Conference on Harmonisation and was approved by the local ethical committee (protocol no. H-17008918) and the data protection officer of the Capital Region of Denmark (journal no. 2012-58-0004). All patients provided written informed consent.

### 2.2. Study Design

The inclusion FDG-PET/CT was defined as the scan at which PsPD was suspected (PsPD FDG-PET/CT). A DTP FDG-PET/CT was acquired within 14 days after the PsPD FDG-PET/CT. Also, a follow-up scan (FU FDG-PET/CT) was done two to three months later (median 2.6 months (IQR; 1.87–2.8)) to determine whether the suspected PsPD was indeed PD or confirmed PsPD (including patients with stable disease, partial or complete response at the follow-up scan).

Furthermore, the FDG-PET/CT scan performed prior to the PsPD FDG-PET/CT was retrieved (median 2.9 months (IQR; 2.73–3.17)), and the two scans were compared using PERCIMT.

### 2.3. FDG-PET/CT

Plasma blood glucose, body weight, and body height were measured before intravenous injection of a standard dose of FDG (4 MBq/kg body weight). The patient was fasting for four hours before injection of the tracer. PET images were acquired after 60 min as standard at inclusion and follow-up scans. For DTP FDG-PET/CT an additional scan was acquired after 180 min rest [17].

Images were acquired using a dedicated PET/CT scanner (Biograph mCT 64^®^; Siemens Healthcare, Erlangen, Germany).

### 2.4. Image Analysis

Image fusion of the PET scan and the CT scan, visual assessment, and all measurements were performed with SyngoVia (Simens, Erlangen, Germany) and MIM (MIM software Inc., Cleveland, OH, USA).

### 2.5. FU FDG-PET/CT

The true response was determined in a multidisciplinary conference setting by combining the FU FDG-PET/CT acquired two to three months (median 2.6 months (IQR; 1.87–2.8)) after the PsPD FDG-PET/CT and information on the clinical condition of the patients provided by an oncologist.

### 2.6. DTP FDG-PET/CT

Among the lesions suspected of malignancy on the PsPD FDG-PET/CT, the three most FDG-avid lesions were selected on the early scan (60 min) at the DTP FDG-PET/CT by visual assessment, and the lesions were delineated creating volumes of interest (VOIs). The VOIs were copied to the delayed acquisition scan (180 min). Standard uptake value (SUV)max, SUVmean, and SUVpeak were measured in each VOI on both the early and delayed acquisition. For each SUV measure, the VOI with the highest value was selected for further analysis on both the early and delayed acquisition.

Retention index (RI), defined as the relative change in SUV between early and delayed FDG-PET/CT, was calculated by the following mathematical formula [10]:

RI = (SUVdelayed − SUVearly)/SUVearly) × 100%.

RI was calculated for each SUV measure in the selected VOIs, giving a total of six RI values. If the patient had only two lesions, these two were used. A cut-off of 10% was used as an indication for malignancy [10].

Patients were classified as having PsPD using DTP FDG-PET/CT (PsPD_DTP_) or PD_DTP_ depending on the evaluation on the FU FDG-PET/CT.

### 2.7. PERCIMT

**Figure 1:**
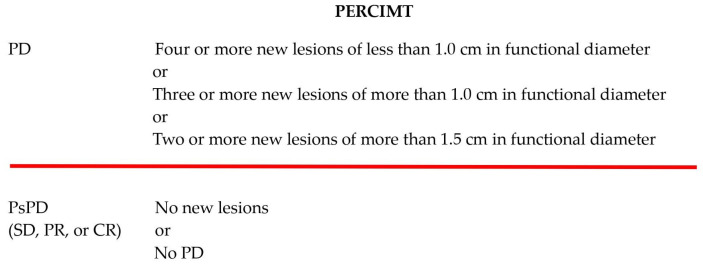
Definition of PERCIMT [12]; PD = progressive disease, SD = stable disease, PR = partial regression, CR = complete regression.

The PERCIMT criteria were applied retrospectively by comparing the PsPD FDG-PET/CT scan with the routine FDG-PET/CT prior to the PsPD FDG-PET/CT. The functional diameter of new lesions on the PsPD FDG-PET/CT was measured, and the response was categorized into either PsPD_PERCIMT_ or PD_PERCIMT_. PsPD_PERCIMT_ includes patients with either stable metabolic disease, or partial or complete metabolic response at the PsPD FDG-PET/CT, whereas PD_PERCIMT_ includes patients with progressive disease, defined as two or more new lesions, taking the size of lesions into account (Figure 1) [12].

### 2.8. Statistics

*p*-values below 0.05 were considered significant. Data management and statistics were performed in the R programming language (version 4.2.3).

Patient characteristics were summarized using percentages or medians and interquartile range (IQR). The maximum, peak, and mean RI values were compared between the true PsPD group and the true PD group using students *t*-test. This was done both for the early and the delayed measurements. A COX regression analysis was used to investigate whether OS and progression-free survival (PFS), respectively, were statistically associated with the response to treatment in both patient groups. The difference in OS and PFS, respectively, was visualized in Kaplan–Meier plots using the R-package “Survminer”, (6 July 2023).

The sensitivity, specificity, positive predictive value (PPV), and negative predictive value (NPV) for DTP FDG-PET/CT and PERCIMT were calculated.

## 3. Results

**Figure 2:**
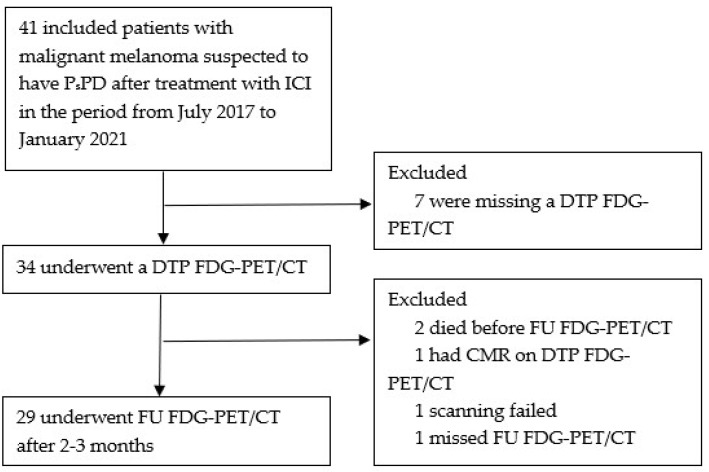
Flowchart; ICI = immune checkpoint inhibitors. PsPD = pseudoprogression. DTP FDG-PET/CT = Dual-time-point fluorodeoxyglucose positron emission tomography/computed tomography. FU FDG-PET/CT = follow-up FDG-PET/CT. CMR = complete metabolic response.

A total of 41 patients were included from July 2017 to January 2021. Of these, 12 patients were excluded due to missing DTP FDG-PET/CT scan (7 patients), death before the FU FDG-PET/CT (2 patients), complete metabolic response on DTP FDG-PET/CT (1 patient), failed scanning (1 patient), or missed FU FDG-PET/CT scan (1 patient) (Figure 2).

Of the 29 evaluable patients, the median age was 69 (IQR; 58–78) years, 52% were male, and 79% had a performance status of 0. All but one patient had stage IV disease (one patient with inoperable stage IIIC), with 18 patients (62%) having M1c disease according to AJCC 8th edition [18]. Almost half of the patients (48%) had three or more organs involved. Nineteen patients (66%) had ≥three metastatic lesions. Most of the patients were treated with anti-PD-1 inhibitors (86%), two (7%) were treated with ipilimumab, and two (7%) with the combination of ipilimumab and nivolumab. Five patients (17%) had received previously treatment lines of ICI (Table 1), and two (7%) were previously treated with BRAF-MEK inhibitors.

Among the 29 patients, 10 (34%) had true PsPD and 19 (66%) had confirmed PD on the FU FDG-PET/CT determined in a multidisciplinary conference setting.

### 3.1. DTP FDG-PET/CT

**Figure 3:**
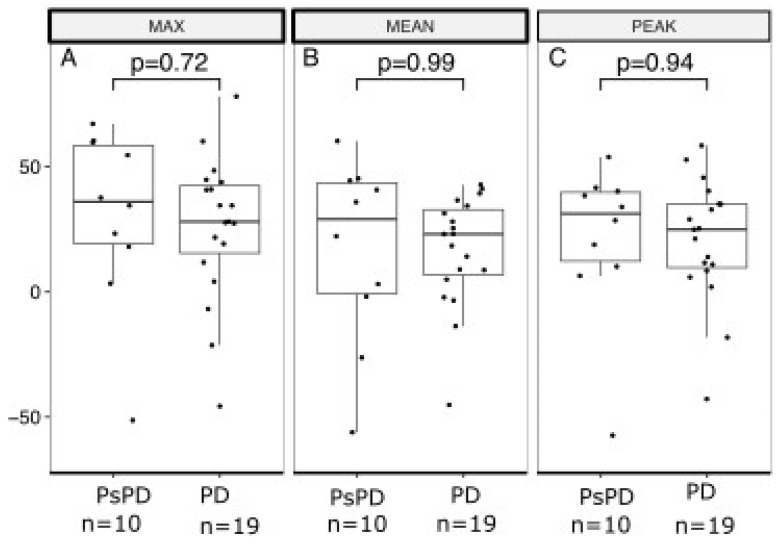
Boxplot of retention index based on early acquisition scan in the true PsPD group versus true PD group. Retention index (RI; *y*-axis) separated between the PsPD group and the PD group (*x*-axis). Students *t*-tests were used to evaluate the significance of the difference for (**A**) MAX, (**B**) MEAN, and (**C**) PEAK. Boxplots indicate the median (central horizontal line) and the lower (25% quantile), and upper (75% quantile) of the data. PsPD = pseudoprogression. PD = progressive disease.

**Figure 4:**
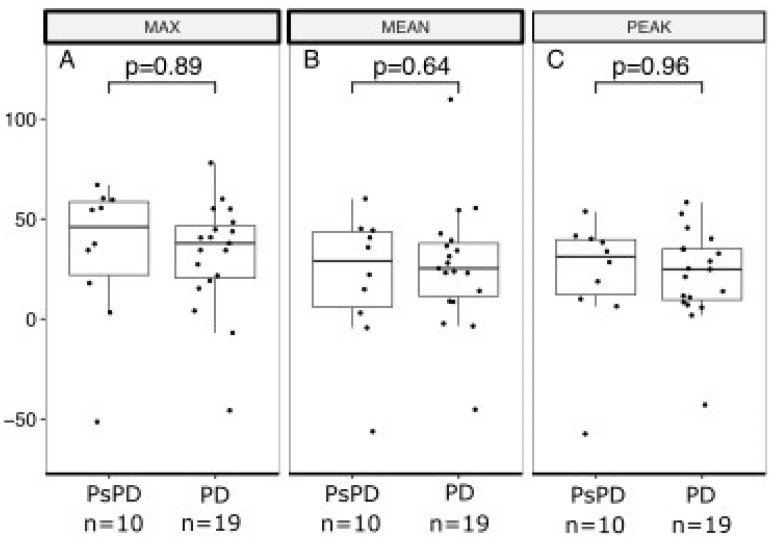
Boxplot of retention index based on delayed acquisition scan in the true PsPD group versus the true PD group. Retention index (RI; *y*-axis) separated between the PsPD group and the PD group (*x*-axis). Students *t*-tests were used to evaluate the significance of the difference for (**A**) MAX, (**B**) MEAN, and (**C**) PEAK. Boxplots indicate the median (central horizontal line) and the lower (25% quantile), and upper (75% quantile) of the data. PsPD = pseudoprogression. PD = progressive disease.

Figure 3 and Figure 4 show boxplots of RI based on the early and delayed scans of the DTP FDG-PET/CT for SUVmax, SUVmean, and SUVpeak. For the early scans, there were no significant differences between the true PsPD group and the true PD group in terms of RImax (*p* = 0.72; Figure 3A), RImean (*p* = 0.99; Figure 3B), or RIpeak (*p* = 0.94; Figure 3C). Likewise, for the delayed scans, we did not observe significant differences in RImax (*p* = 0.89; Figure 4A), RImean (*p* = 0.64, Figure 4B), or RIpeak (*p* = 0.96, Figure 4C).

Using DTP FDG-PET/CT, 7 patients (24%) were classified as having PsPD_DTP_, and 22 patients (76%) were classified as having PD_DTP_.

**Figure 5:**
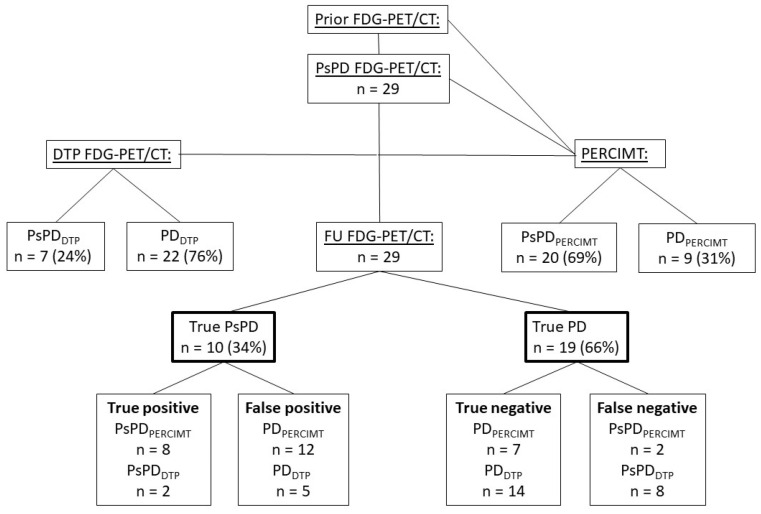
Diagram with an overview of the scans and methods applied and the outcome; FDG-PET = fluorine-18-fluorodeoxyglucose-positron emission tomography. CT = computed tomography. PsPD = pseudoprogression. PD = progressive disease. DTP = Dual-time point. PERCIMT = PET Response Evaluation Criteria for Immunotherapy. FU FDG-PET/CT = follow-up FDG-PET/CT. PsPD FDG-PET/CT = The inclusion FDG-PET/CT with suspected PsPD.

Among the 10 patients with true PsPD, DTP FDG-PET/CT detected 2 patients (true positive), resulting in a PPV of 29%. Out of the 19 patients with true PD, the DTP FDG-PET/CT scan identified 14 patients (true negative), with NPV of 64% (Figure 5).

The sensitivity and specificity of the DTP FDG-PET/CT were 20% and 74%.

### 3.2. PERCIMT

Twenty patients (69%) were classified as having PsPD_PERCIMT_, and nine patients (31%) were classified as having PD_PERCIMT_. Eight patients were true positive resulting in a PPV of 40%, and seven patients were true negative, with NPV of 78% (Figure 5).

The sensitivity and specificity when applying PERCIMT to the FDG-PET/CT scans were 80% and 37%.

### 3.3. Survival Outcome

At the time of this analysis, 22 of 29 patients (76%) had died, with a median OS of 22 months (95% CI, 18–54). Median FU was five months (95% CI, 2–60).

**Figure 6:**
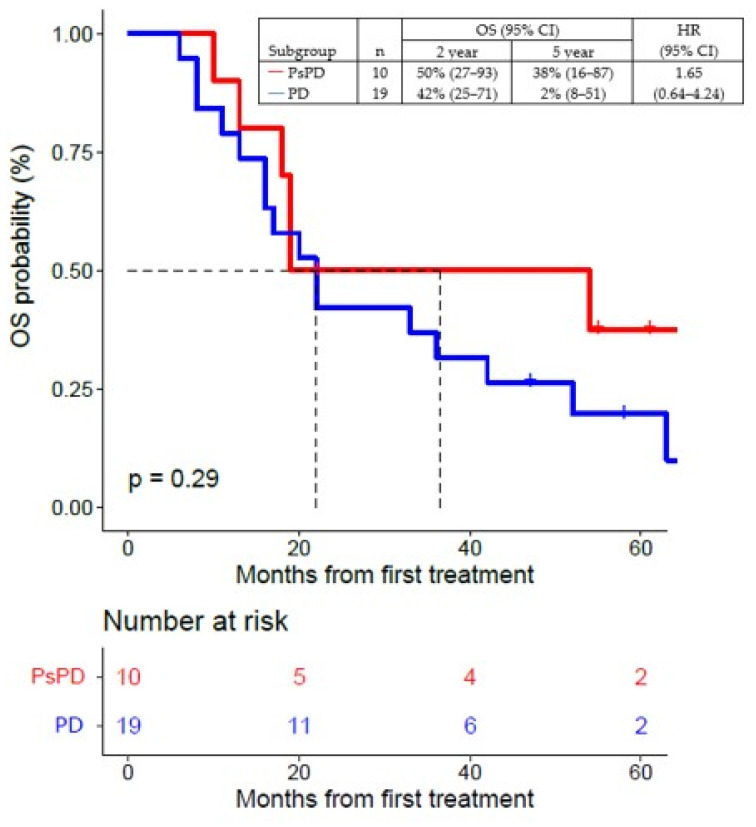
Overall survival (OS) of patients with true PsPD versus patients with true PD; statistical significance (*p* value) has been evaluated using a COX regression. PsPD = pseudoprogression. PD = progressive disease. OS = overall survival. HR = hazard ratio.

The 2-year OS rate was 50% (95% CI, 27–93), and the 5-year OS rate was 38% (95% CI, 16–87) in the group of patients with true PsPD, and, respectively, 42% (95% CI, 25–71) and 2% (95% CI, 8–51) in patients with true PD (Figure 6).

**Figure 7:**
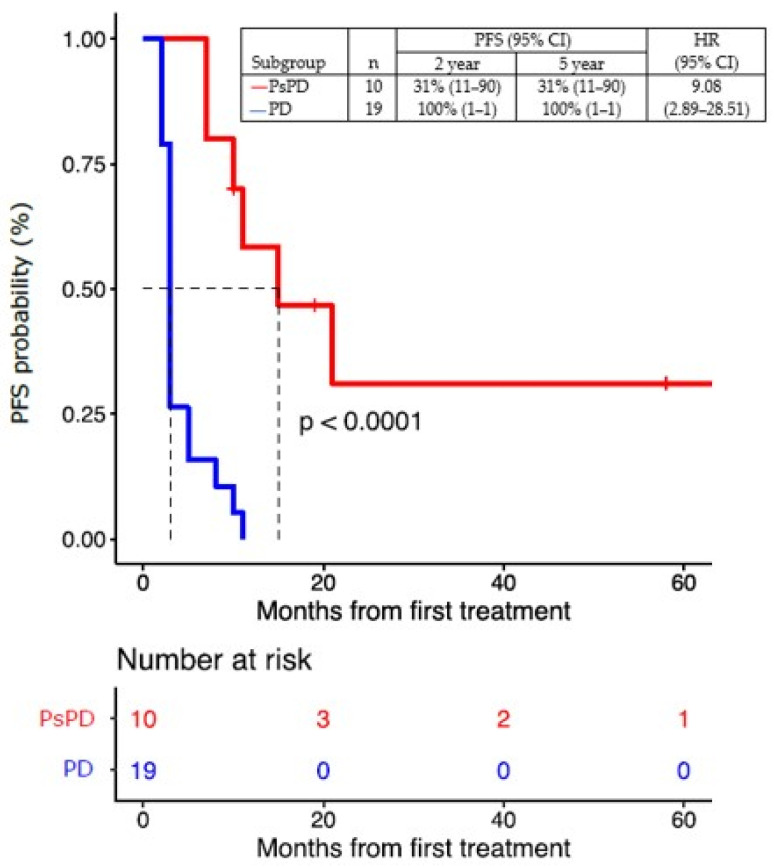
Progression-free survival (PFS) of patients with true PsPD versus patients with true PD. Statistical significance (*p* value) has been evaluated using a COX regression. PsPD = pseudoprogression. PD = progressive disease. PFS = progression-free survival. HR = hazard ratio.

PD or death had occurred in 27 of 29 patients (93%) at the time of this analysis. The median PFS for all patients was five months (95% CI, 3–10), with a 2-year PFS rate of 31% (95% CI, 11–90) and a 5-year PFS rate of 31% (95% CI, 11–90) in patients with true PsPD. All patients progressed as per definition in the group of patients with true PD (Figure 7).

## 4. Discussion

This prospective study is the first to investigate the use of DTP FDG-PET/CT to detect PsPD. This is based on knowledge from previous studies demonstrating that the uptake of FDG continues to increase in malignant tumors for several hours after the injection of the radiotracer. In this study, we investigated two FDG-PET-guided methods to increase the sensitivity in the detection of PsPD in patients with metastatic melanoma treated with ICI.

In a retrospective evaluation, only 10 out of 29 (34%) patients suspected of PsPD were found to have true PsPD, while 19 patients (66%) were defined as having confirmed PD. Using DTP FDG-PET/CT, we were only able to identify 2 out of the 10 patients having true PsPD, and 14 out of the 19 patients with confirmed PD showed poor sensitivity and specificity (20% and 74%). Applying PERCIMT criteria, we identified 20 patients with PsPD, which included 8 out of the 10 patients with true PsPD; and, furthermore, we identified 9 patients with PD using PERCIMT, which included 7 out of the 19 patients with true PD, showing a sensitivity of 80% and a specificity of 37%.

In this explorative analysis, we only included patients clinically suspected to have PsPD, and it is therefore not possible to determine the overall sensitivity and specificity of the FU FDG-PET/CT. Neither the PsPD FDG-PET/CT, the DTP FDG-PET/CT, or application of PERCIMT appeared to be able to diagnose PsPD with sufficient accuracy. PsPD could only be diagnosed retrospectively, and only in a multidisciplinary meeting which was done according to European guidelines [21].

The study has multiple limitations. The pre-test probability of PsPD was high, as it was only possible to include patients suspected of PsPD due to limited scanner capacity, and the fact that DTP FDG-PET/CT is time consuming for both the patients and the staff. Furthermore, the cohort was small, and unfortunately many of the included patients did not have the DTP FDG-PET/CT scan due to various reasons, resulting in exclusion of these patients and an even PERCIMTer patient cohort. This complicates the ability to see trends and to make firm conclusions on the results, especially in the survival analyses. Furthermore, PERCIMT was developed for other treatment regimens, and therefore was not designed to the patient group of this study; and, moreover, our population had suspected PsPD at inclusion [12]. However, we found, in accordance with the meta-analysis by Ayati et al. [13], PERCIMT to be the most promising criteria to diagnose PsPD.

Other strategies to identify PsPD in the case of progression according to Response Evaluation Criteria in Solid Tumors (RECIST [22]) during treatment with ICI should be investigated. Kong et al. [23], analyzed biopsies from patients treated with ICI after a mean of 15.2 months and found that three out of eight patients with existing positive FDG-PET/CT lesions had immune cell infiltrates and not melanoma. Tumor biopsies at the time of progression could potentially identify patients with a high degree of tumor infiltrating lymphocytes, but this is an invasive procedure, reducing its feasibility as a standard. Also, it has not yet been determined which degree of tumor infiltration that would translate into clinical response, and finally, this procedure and investigation might take just as long as the time to perform a new evaluation scan and conclude clinically whether the patient is still progressing or had a late response.

As another strategy, many ongoing trials have included the use of circulating tumor DNA (ctDNA), but the advantages have especially been shown in other cancers, i.e., colorectal cancer [24,25]. However, Lee et al. [26] have investigated whether ctDNA can differentiate between the radiologic findings of PsPD and true PD in patients with metastatic melanoma treated with ICI. Of 29 patients, 9 (31%) had PsPD. Sensitivity of ctDNA for predicting PsPD was 90% (95% CI, 68–99) and specificity was 100% (95% CI, 60–100). Thus, measurement of ctDNA might represent a less invasive and more valid future in the evaluation of PsPD.

Also, other PET tracers like 68Ga FAPI 18FLT, 11C-MET, or immuno-PET radiotracers targeting T-cells or immune checkpoint targets, may play a future role in the evaluation of response in melanoma [27,28,29,30].

## 5. Conclusions

This study indicates that DTP FDG-PET/CT exhibits limited efficacy in differentiating between PsPD and true PD. The results of this exploratory analysis do not support further investigation of DTP FDG-PET/CT, and its use is not recommended in the evaluation of patients suspected of having PsPD. Furthermore, PERCIMT criteria should be used only in accordance with clinical assessment and, if possible, in a multidisciplinary team conference taking the treatment regimen into consideration. Therefore, we suggest that treatment with ICI should be continued in cases where PsPD is suspected, limited immunotoxicity is observed, and no major clinical signs of progression exist, until a FU FDG-PET/CT can be obtained, aligning with current clinical practice.

## Figures and Tables

**Table 1:** Baseline demographics and characteristics.

	**n = 29**
**Age, years**	69 (58–78)
**Sex**	
Male	15 (52%)
Female	14 (48%)
**ECOG performance status**	
0	23 (79%)
1	5 (17%)
2	1 (3%)
**LDH concentration**	
<Upper limit of normal	14 (48%)
≥Upper limit of normal	15 (52%)
**Tumor stage**	
IIIC	1 (3%)
IV M1a	4 (14%)
IV M1b	6 (21%)
IV M1c	18 (62%)
**Number of organs involved**	
<3	15 (52%)
≥3	14 (48%)
**BRAF mutation status**	
Mutation	10 (34%)
Wildtype	19 (34%)
**Melanoma diagnosis**	
Cutaneous	21 (72%)
Melanoma of unknown primary	8 (28%)
**Immune checkpoint inhibitors**	
anti-PD-1 inhibitors *	25 (86%)
Ipilimumab	2 (7%)
Ipilimumab + Nivolumab	2 (7%)
**Previous immune checkpoint inhibitors**	
anti-PD-1 inhibitors **	4 (14%)
Ipilimumab	1 (3%)
Ipilimumab + Nivolumab	1 (3%)

Data are median (interquartile range) or n (%). ECOG = Eastern Cooperative Oncology Group. LDH = lactate dehydrogenase. PD-1 = programmed death 1. * Include pembrolizumab (20 patients), nivolumab (4 patients), or protocol (nivolumab + an IDO/PD-L1 peptide vaccine [19]) (1 patient). ** Include pembrolizumab (3 patients) or RELATIVITY-047 (LAG3 + nivolumab [20]) (1 patient).

## Data Availability

The data that support the findings of this study can be made available upon reasonable request.
